# Nonadherence to oral cancer chemotherapy in hepatocellular carcinoma: prevalence and predictive factors in Vietnam

**DOI:** 10.1186/s12885-024-12601-2

**Published:** 2024-07-15

**Authors:** Thai Doan Ky, Nguyen Thi Loan, Nguyen Tien Thinh, Mai Thanh Binh

**Affiliations:** Department of Gastroenterology and Hepatology, 108 Military Central Hospital, Hanoi, Vietnam

**Keywords:** Hepatocellular carcinoma, Outpatients, Oral chemotherapy, Nonadherence

## Abstract

**Purpose:**

Standard oral cancer chemotherapy (OCT) or targeted therapy (OTT) has expanded the treatment methods for hepatocellular carcinoma (HCC). However, its principal nonadherence causes a reduction in efficacy. We aimed to evaluate the status of nonadherence and influencing factors among outpatient patients with HCC.

**Patients and methods:**

In 2021, a prospective observational study was conducted on 384 patients with either old or newly diagnosed HCC treated with OTT. Nonadherence to OCT was determined using the eight-item Morisky Medication Adherence Scale, with a score < 6 points. The patients were finished with a six-month follow-up investigation by questionnaires.

**Results:**

54,8% of HCC outpatients were nonadherent to OCT, with a mean Morisky score of 5.19. They dropped out of the treatment mainly because of drug side effects, such as fatigue (72.4%), hand-foot syndrome (42.7%), diarrhea (38.3%), nausea (25%), insomnia (24.7%), abdominal pain (12%), and anxiety about these adverse events (65.9%). Additionally, financial difficulties and low relative copayments were significantly correlated with the noncompliant treatment of patients (OR = 2.29, 95% CI = 1.32–3.98, *P* = 0.003; OR = 4.36, 95% CI = 0.95–19.93, *P* = 0.039, respectively). Moreover, inadequate individual information about the clinical course, the art of treatment, and medication usage instructions were suggestive barriers to adherence to treatment (OR = 1.96, 95% CI = 1.08–3.55, *P* = 0.024; OR = 1.86, 95% CI = 1.1–3.14, *P* = 0.02; OR = 2.34, 95% CI = 1.29–4.26, *P* = 0.004, respectively). Finally, a low level of trust in doctors was an essential factor in nonadherence (Mean of the Anderson Trust in Physician Scale scores counted 38.12 vs. 43.97, respectively for non-adherence vs. adherence, *P* = 0.00001).

**Conclusions:**

This study suggests a high rate of primary nonadherence to standard oral targeted therapy among HCC outpatient patients because of drug side effects, patient awareness of treatment, and lack of confidence in healthcare providers. Close supervision, proper medication instructions, appropriate dosage reductions, and comprehensive patient counseling might be necessary to control nonadherence.

## Introduction

Liver cancer is one of the most common cancers in Asia in terms of incidence and mortality, accounting for nearly three-quarters of all liver cancer cases worldwide. Hepatocellular carcinoma (HCC) is the predominant primary neoplasm in liver cancers. Hepatitis B and C viruses are the primary pathogens that cause HCC. In Vietnam, HCC is the most dangerous cancer, with the highest rates of both new diagnoses and deaths of patients [1]. This HCC status in Vietnam is due to late diagnosis of HCC at baseline. Hepatectomy is the cornerstone of curative options for patients with early-stage HCC globally [2]; however, most Vietnamese patients are diagnosed at an advanced stage [3], which is associated with poor clinical outcomes [1, 3]. For the treatment of late-stage HCC, sorafenib was first approved by the FDA in 2005 [4] and by the Vietnamese government in 2009 [5]. Until now, sorafenib has remained the first line of treatment in the intermediate and advanced stages of HCC [5, 6].

Adherence to standard oral cancer chemotherapy (OCT) or targeted therapy (OTT) has played an essential role in the successful treatment of many cancer patients; however, the rate of nonadherence remains high [7, 8]. According to the World Health Organization, medication adherence is defined as “the extent to which a person’s medication-taking behavior corresponds with the agreed recommendations from a healthcare provider.” Medication adherence is a multidimensional issue influenced by five factors: patient-related factors, complex regimens, disease conditions, the healthcare system, and socioeconomic factors [9, 10]. Among patients with HCC, low adherence to oral antineoplastic agents, such as sorafenib, is mainly caused by forgetfulness and side effects, which result in worse clinical outcomes [11, 12]. There were a few reports worldwide indicating a low adherence rate to cancer drug treatment in HCC patients. Author Sujia reported that the adherence rate to treatment in kind of patients with a moderate or higher level reached only 56%, with a mean Morisky scale score of 5.44 [13]. They demonstrated a low rate of adherence to oral chemotherapy due to the cost of medication, side effects, and lack of accessibility to health information and social support [13]. A notable correlation was observed between non-adherence and factors such as body mass index, potassium level, albumin, and medication costs [13].

In Vietnam, Sorafenib has been the first-choice treatment for HCC since 2009 and is partly covered by social insurance. Other prescribed oral cancer medication regimens, such as Lenvatinib, Regorafenib, and Cabozantinib, have been used for the treatment of HCC without coverage from health insurance but receive partial support from pharmaceutical companies [5]. However, data on their adherence remains limited. Therefore, we studied the status and factors associated with low adherence among patients with HCC treated with oral antineoplastic therapies. Clarifying the factors contributing to the significant increase in non-adherence to targeted drug treatment in these patients will contribute to better guidance, communication, and support from clinical physicians for patients during the medication use process. This assists in enhancing the effectiveness of the treatment approach, providing more positive value for liver cancer patients.

## Materials and methods

### Patients

We randomly recruited 384 Vietnamese adult outpatients with HCC at 108 Military Central Hospital, Hanoi, Vietnam, between January 2021 and August 2021.

Inclusion criteria: Adult Patients (≥ 18 years old) with intermediate- and advanced-stage HCC who newly or formerly received OCT, including Sorafenib, Lenvatinib, and Regorafenib, visited the hospital in an appointment with doctors. They had good performance status (ECOG 0,1) and compensated liver function (Child-Pugh A or B 7 points). They were finished with a six-month follow-up investigation by questionnaires.

Exclusion criteria: Patients who received immunotherapy concomitantly, disagreed to participate in the study or did not complete the questionnaire were excluded.

### Ethics statement

Written informed consent was obtained from all study participants after a detailed explanation of the study was provided at the time of the survey. The Institutional Review Board of 108 Military Central Hospital, Hanoi, Vietnam, approved the study protocol. All data were analyzed in accordance with the guidelines and regulations related to data security.

### Measurement

**Fig. 1:**
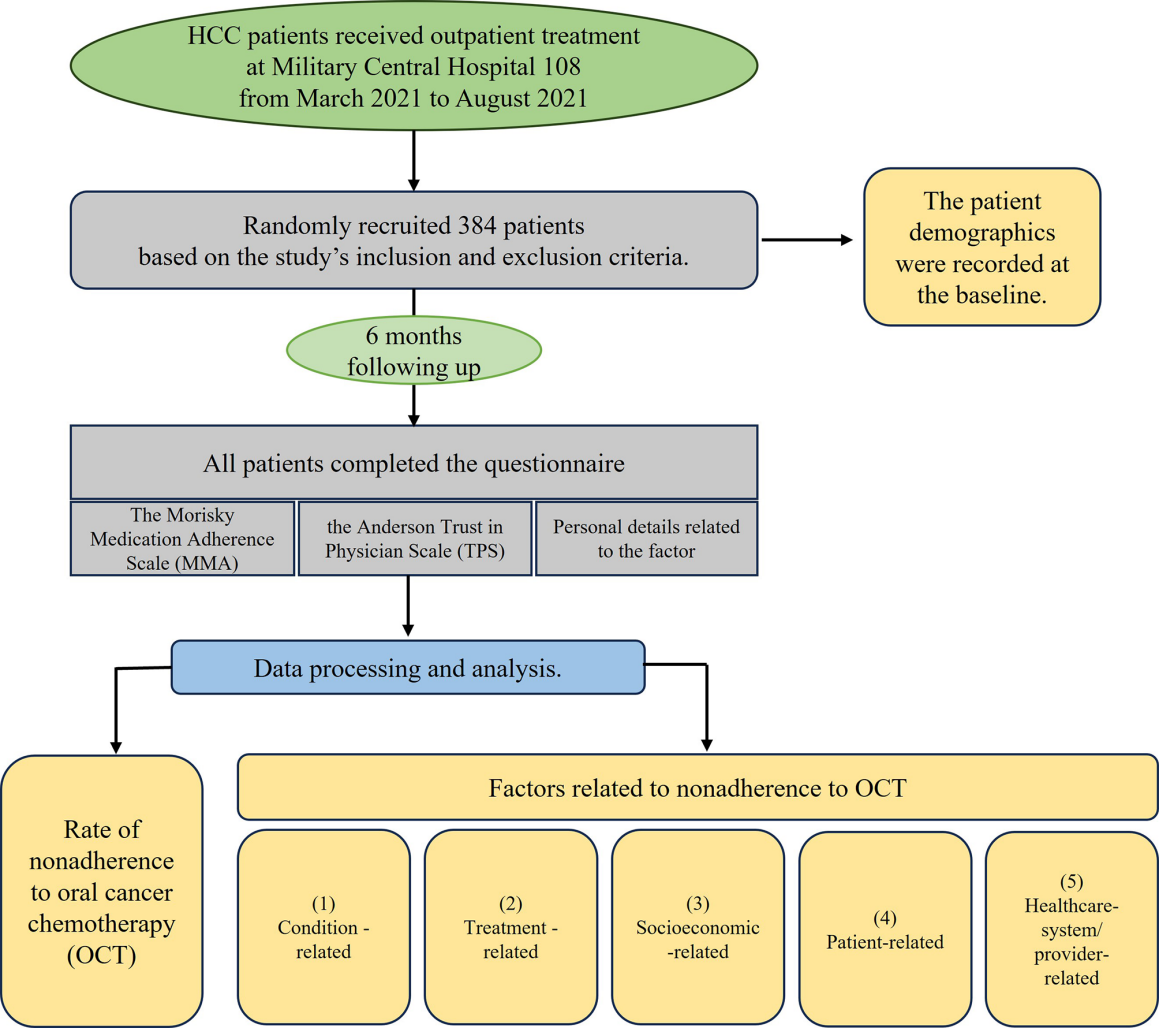
Study framework

A prospective observational study was conducted on 384 HCC patients, with a questionnaire after six months following up (Fig. 1). The patient demographics were recorded, including marital status, insurance status, medical comorbidities, background knowledge, laboratory studies, and the dose of OTC drug at the baseline. The medication dosage used to analyze treatment non-adherence was the amount of TKI medication the patient took, recorded at the baseline. This could be the high dose for newly prescribed TKIs or the initial dose / the adjusted dose for patients already undergoing treatment. A high dose refers to the maximum recommended dose of the specific TKI, such as Sorafenib 800 mg/day, Lenvatinib 12 mg/day, and Regorafenib 160 mg/day.

All patients completed the questionnaire, including the Morisky Medication Adherence Scale (MMAS) [14], the Anderson Trust in Physician Scale (TPS) [15], and personal details related to the factors affecting adherence.

The MMAS contains eight self-reported items with seven yes or no questions, and the last question is answered on a 5-point Likert scale (reliability = 0.83). Each ‘‘no’’ answer yielded one point, and the last question was scored as 0, 0.25, 0.5, 0.75, or 1 [14–16]. A score < 6 points was classified as “nonadherence.”

The factors of nonadherence were identified as WHO, including (1) Condition-related, such as comorbidities, stages of HCC and Child-Pugh score; (2) Treatment-related, including a regiment of medication and side effects of the drugs (The information on the side effects of the patient’s targeted medication was obtained through direct reporting from the patient to their attending physician. This communication occurred via messages, phone calls, and image submissions. Subsequently, the physicians assessed the extent of the patient’s medication side effects and guided the management of discomfort symptoms. Additionally, details about encountered side effects were documented in the questionnaire at the patient’s follow-up after 3 or 6 months); (3) Socioeconomic-related, including occupation, education (The education level was scored based on the attainment of a degree from a university, with “high education” defined as a degree from university or higher, and conversely as “low education”), and financing (Economic difficulties of patients were identified when their income is below 400 USD/month. “Relative co-payment” refers to economic support from siblings within the family, and “Co-payment from friends/neighbors” represents support from the patient’s friends, neighbors, or community welfare funds); (4) Patient-related, like age, gender, patient’s awareness of the illness and their corresponding knowledge background (The knowledge background of patients included their understanding about “the status of the diseases” like liver stages and the reasons they were prescribed oral cancer medication; “the art of treatment”, which referred the comprehensive strategy for systemic drug therapy that a patient would undergo; and “the medication usage instruction”, which referred to how to use the medication during the day, the quantity of pills, the timing of taking the medication, whether it should be taken before or after meals, etc.) (5) Healthcare-system/provider-related such as level of trust in doctor [9, 10] (The level of trust in doctors was evaluated using the Anderson Trust in Physician Scale (TPS) with 11 questions, each rated on a 5-point Likert scale (1–5 points) [17]).

### Statistical analysis

Patients were classified as nonadherent if they scored < 6 on the MMAS. The prevalence of non-adherence in HCC who received OCT was calculated as the proportion of patients within the study group who did not adhere to the treatment.

Differences in baseline characteristics according to medication adherence status were analyzed using chi-square for categorical variables. Chi-square test ***(χ***^***2***^**)** and Fisher’s exact tests were utilized to assess the factors of nonadherence to oral chemotherapy. An Independent t-test was used to compare the mean, given the mean of the Anderson Trust in Physician Scale (TPS), between the 2 groups.

All statistical analyses were performed using SPSS version 22 software (IBM Corp., Armonk, NY, USA). Statistical significance was set at *P* < 0.05.

## Results

### Baseline characteristics of hepatocellular carcinoma patients

The demographic characteristics of 384 patients with study participants are summarized in Table 1. Of the 384 patients, 354 (92%) were male and 30 (8%) were female. The mean patient age was 60 (26–86 years). Among them, 84% had chronic HBV infection, 5% had chronic HCV infection, and 35% consumed alcohol. Moreover, 97% of patients were married. Although 98% of patients had cirrhosis, most had compensation for liver function.

**Table 1:** Characteristics of 384 outpatient Vietnamese HCC patients at baseline

Characteristics	Total (*n* = 384)	Adherence (*n* = 166)	Non-adherence (*n* = 218)	*P* Value
Age (years)	60 (26–86)			
Age ≥ 60 years old (n, %)	234 (60.9%)	99 (59.6%)	135 (61.9%)	0.6
Male (n, %)	354 (92%)	154 (92.8%)	200 (91.7%)	0.7
BMI < 23 (n, %)	355 (92%)	156 (94%)	199 (91.3%)	0.3
Married (n, %)	371 (97%)	160 (96.4%)	211 (96.8%)	0.8
*Etiology of liver disease*				
HBV (n, %)	323 (84%)	138 (83.1%)	185 (84.9%)	0.6
HCV (n, %)	21 (5%)	8 (4.8%)	13 (6%)	0.6
Alcohol (n, %)	135 (35%)	120 (72.3%)	160 (73.4%)	0.8
*Comorbidities*				
Cardiovascular diseases (n, %)	36 (9%)	16 (9.6%)	20 (9.2%)	0.8
Respiratory diseases (n, %)	132 (34%)	52 (31.3%)	80 (36.7%)	0.2
Cirrhosis (n, %)	377 (98%)	161 (97%)	216 (99.1%)	0.1
*Child-Pugh classification*				
Child-Pugh A (n, %)	351 (91%)	156 (94%)	195 (89.4%)	0.7
Child-Pugh B (n, %)	33 (9%)	10 (6%)	23 (10.6%)	
*BCLC staging*				
BCLC B (n, %)	137 (36%)	68 (41%)	69 (31.7%)	
BCLC C (n, %)	247 (64%)	108 (65.1%)	139 (63.8%)	0.3

Values given are medians or numbers and percentiles where appropriate. n, numbers of individuals; BCLC: Barcelona Clinic Liver Cancer. *P* values were calculated by the Chi-square test

### Rate of nonadherence to medicine

Overall, based on the MAA survey, 208 (54.8%) patients were defined as nonadherent (with mean Morisky scores of 5.19 ± 0.52), and 176 (45.2%) were adherers who even had low mean Morisky scores (6.82 ± 0.45) (Table 2). Moreover, among all patients, 43.8% said that they “sometimes forgot to take their medication, and 33.9% reported that they cut back or stopped taking their medicine without telling their doctor because they felt worse when they took it. It is noteworthy that half of the patients forgot to take their medication the day before their appointments, and one-fifth of the patients did not take the drug for two weeks before the appointment (Table 3).

**Table 2:** Rate of adherence and non-adherence in standard oral cancer chemotherapy

	*n*	%	Mean ± SD
**Non-adherer**	208	54.8	5.19 ± 0.52
**Adherer**	176	45.2	6.82 ± 0.45

The mean given is the mean of Morisky Medication Adherence Scale scores, which contained eight self-reported items with seven yes or no questions and a last question answered on a 5-point Likert scale (a reliability = 0.83). Each ‘no’ answer yielded one point, and the previous question was calculated as 0, 0.25, 0.5, 0.75, and 1. Values given are numbers and percentile

**Table 3:** The 8-item Morisky Adherence questions

Questions			No (*n*=)	% of No
1.	Do you sometimes forget to take your medicine?		168	43.8
2.	People sometimes miss taking their medicines for reasons other than forgetting. Thinking over the past two weeks, were there any days when you did not take your medicine?		256	66.7
3.	Have you ever cut back or stopped taking your medicine without telling your doctor because you felt worse when you took it?		130	33.9
4.	When you travel or leave home, do you sometimes forget to bring along your medicine?		42	10.9
5.	Were there any medications you did not take yesterday?		190	49.5
6.	When you feel like your symptoms are under control, do you sometimes stop taking your medicine?		3	0.8
7.	Taking medicine every day is a real inconvenience for some people. Do you ever feel hassled about sticking to your treatment plan?		16	4.2
8.	How often do you have difficulty remembering to take all your medicine?	+ Never/rarely	40	10.4
		+ Once in a while	138	35.9
		+ Sometimes	114	29.7
		+ Usually	40	10.4
		+ All the time	52	13.5

The total percentage of No answers to each question is reported

When stratified into subgroups (new TKI users and ongoing TKI users), our analysis did not reveal any significant differences in treatment adherence to TKIs (data not shown).

### Association between diseases condition related factors and nonadherence

The patient’s disease status, either HCC, including etiology of liver disease, the liver cancer stages (classified by BCLC), and liver function (according to the Child-Pugh score) or comorbidities, was not correlated with the patient’s treatment adherence (Table 1).

### Therapy-related factors correlated with nonadherence to OCT

**Fig. 2:**
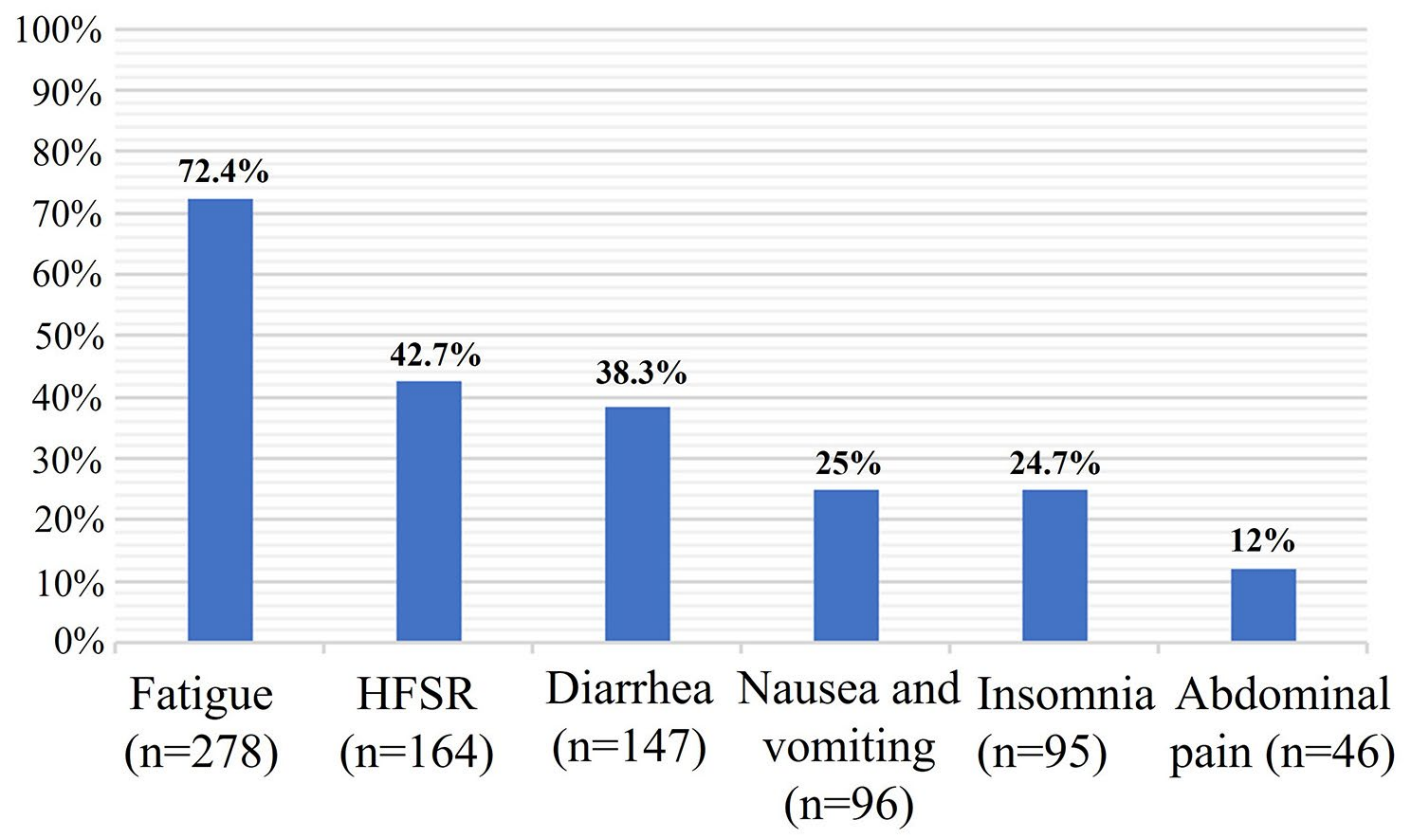
Treatment-emergent adverse events. Each adverse event was given by the number of cases and percentage of 384 HCC patients who received oral cancer chemotherapy. HFSR, Hand-foot skin reaction

The overall side effects of oral antineoplastic therapies are shown in Fig. 2. Patients experienced the most adverse effects, fatigue (72.4%), followed by hand-foot syndrome, diarrhea, nausea and vomiting (42.7%, 38.3%, and 25%, respectively). Of all individuals, 24.7% suffered from insomnia, and 12% experienced abdominal pain. Notably, all these effects were reported with a significantly higher frequency among nonadherent individuals than among adherent individuals (Table 4), which indicated that common side effects of prescribed oral medication regimens correlate with patient adherence. In addition, different regimens of oral antineoplastic therapy and high doses were not found to be related to nonadherence levels (data not shown).

**Table 4:** Association of side effects of the drugs with non-adherence

TEAEs		Adherence		Non - adherence		OR (95% CI)	(χ^2^)	*P* Value
		(*n* = 166)		(*n* = 218)				
		n=	%	n=	%			
Fatigue	No	63	59.4	43	40.6	1		
	Yes	103	37.1	175	62.9	2.49(1.58–3.93)	**15.7**	**< 0.0001**
Nausea	No	133	46.2	155	53.8	1		
	Yes	33	34.4	63	65.6	1.64 (1.01–2.65)	**4.1**	**0.043**
Abdominal pain	No	157	46.4	181	53.6	1		
	Yes	9	19.6	37	80.4	3.57(1.67–7.62)	**11.9**	**0.001**
Insomnia	No	138	47.8	151	52.2	1		
	Yes	28	29.5	67	70.5	2.19(1.33–3.60)	**9.7**	**0.002**
HFSR	No	107	48.6	113	51.4	1		
	Yes	59	36	105	64	1.69(1.1–2.55)	**6.1**	**0.013**
Diarrhea	No	122	51.5	115	48.5	1		
	Yes	44	29.9	103	70.1	2.48(1.61–3.84)	**17.2**	**0.0001**

TEAEs, Treatment-emergent adverse events; HFSR, Hand-foot skin reaction; n, numbers of individuals; OR, Odd ratio. Values given are numbers and percentile. OR (95% CI) and *P* values were calculated using Fisher’s exact test. ***(χ***^***2***^**)** was calculated by the Chi-square test

### Factors related to patients’ socioeconomic status linked with nonadherence to OCT

The status related to patients’ finances is summarized in Table 5. Among the 218 nonadherers, 193 (88%) had difficulty with their treatment budgets. As a result, financial challenges play a role in increasing the rate of nonadherence to oral antineoplastic agents (OR = 2.29, 95% CI = 1.32–3.98, *P* = 0.003). Moreover, copayments from relatives and others, such as friends or neighbors, were observed more frequently in the adherence group than in the nonadherence group (OR = 4.36, 95% CI = 0.95–19.93, *P* = 0.039; and OR = 1.76, 95% CI = 1.17–2.65, *P* = 0.007, respectively). There were no differences between the two groups regarding occupation or education.

**Table 5:** The association between patient’s socioeconomic and their non-adherence

Factors from patients		Adherence (*n* = 166)		Non-adherence (*n* = 218)		OR (95% CI)	(χ^2^)	*P* Value
		n=	%	n=	%			
Occupation	Officials	56	44.8	69	55.2	1		
	Freelance	44	45.8	52	54.2	1.04 (0.61–1.78)	0.02	0.88
	Retirements	66	40.5	97	59.5	0.84 (0.52–1.34)	0.54	0.46
Education	High	64	43.5	83	56.5	1		
	Low	102	43	135	57	1.02 (0.67–1.55)	0.09	0.92
Financial difficulties	No	38	63.7	25	39.7	1		
	Yes	128	39.9	193	60.1	2.29 (1.32–3.98)	**8.97**	**0.003**
Relative co-payment	Yes	164	44.2	207	55.8	1		
	No	2	15.4	11	84.6	4.36 (0.95–19.93)	**4.25**	**0.039**
Co-payment from friends/neighbors	Yes	103	49.5	105	50.5	1		
	No	63	35.8	113	64.2	1.76 (1.17–2.65)	**7.32**	**0.007**

n, numbers of individuals; OR, Odd ratio. Values given are numbers and percentile. OR (95% CI) and *P* values were calculated using Fisher’s exact test. ***(χ***^***2***^**)** was calculated by the Chi-square test

### The connection between patient-related factors and adherent rate

We assess the role of patient-related factors, including the patient’s awareness of the illness and their corresponding knowledge background, with nonadherent treatment.

To explore the role of the patient’s awareness of the illness, we compared the frequency of difficulty in medication administration, the awareness of the disease, and concerns about oral cancer treatment drugs. As expected, a high proportion of difficulty and refusal to take oral cancer medication was more frequently observed among nonadherers than among adherers (OR = 1.69, 95% CI = 1.09–2.63, *P* = 0.02; and OR = 1.67, 95% CI = 1.1–2.5, *P* = 0.017, respectively) (Table 6). Moreover, nonadherent patients reported more concern about the side effects of the medication than adherent patients (OR = 2.01, 95% CI = 1.36–3.1, *P* = 0.001) (Table 6).

**Table 6:** The relationship between nonadherence and the patient-related factors

Factors from patients		Adherence (*n* = 166)		Non-adherence (*n* = 218)		OR (95% CI)	(χ^2^)	*P* Value
		n=	%	n=	%			
Difficulty in taking medication	No	123	47.3	137	52.7	1	**5.5**	**0.02**
	Yes	43	34.7	81	65.3	1.69 (1.09–2.63)		
Diminished sense of priority for medication	No	106	48.6	112	51.4	1	**6.0**	**0.014**
	Yes	60	36.1	106	63.9	1.67 (1.1–2.5)		
Anxiety about the side effects of medication	No	104	51.5	98	48.5		**11.8**	**0.001**
	Yes	62	34.1	120	65.9	2.01 (1.36–3.1)		

n, numbers of individuals; OR, Odd ratio. Values given are numbers and percentile. OR (95% CI) and *P* values were calculated using Fisher’s exact test. ***(χ***^***2***^**)** was calculated by the Chi-square test

Similarly, the background of knowledge about clinical HCC courses, treatment of HCC, and medication was also significantly lower among nonadherent individuals (< 30%) than among adherent individuals (~ 70%) (Table 7), suggesting that insufficient understanding related to aspects of HCC is barrier-compliant oral antineoplastic therapies (OR = 1.96, 95% CI = 1.08–3.55, *P* = 0.024; OR = 1.86, 95% CI = 1.1–3.14, *P* = 0.02, and OR = 1.67, 95% CI = 1.1–2.5, *P* = 0.017, respectively).

**Table 7:** The correlation between patients’ background knowledge and non-adherent rates

Understanding the patients about		Adherence (*n* = 166)		Non-adherence (*n* = 218)		OR (95% CI)	(χ^2^)	*P* Value
		n=	%	n=	%			
The status of the disease	Yes	148	45.7	176	54.3	1	**5.07**	**0.024**
	No	18	30	42	70	1.96(1.08–3.55)		
The art of treatment	Yes	141	46.2	164	53.8	1	**5.44**	**0.02**
	No	25	31	54	68.4	1.86(1.1–3.14)		
The medication usage instructions	Yes	149	46.4	172	53.6	1	**8.1**	**0.004**
	No	17	27	46	73	2.34(1.29–4.26)		

The art of treatment includes the treatment methods for each stage, the integration of various treatment approaches, the duration of targeted drug use, when to schedule follow-up assessments, and when to discontinue or switch to other targeted treatment medications. n, numbers of individuals; OR, Odd ratio. Values given are numbers and percentile. OR (95% CI) and *P* values were calculated using Fisher’s exact test. ***(χ***^***2***^**)** was calculated by the Chi-square test

### The correlation between the healthcare-system/provider-related factors and the adherence of patients

Finally, we analyzed the association between the level of trust in doctors and the rate of disobedience (Table 8). Patients who dropped out of the treatment had significantly lower levels of trust in doctors than those who adhered to the treatment (mean Anderson Trust in Physician Scale scores of 38.12 ± 5.41 vs. 43.97 ± 6.05, *P* = 0.0001). This finding indicated that the level of adherence increased with the level of trust in doctors.

**Table 8:** The association between levels of trust in doctors of patients and non-adherent treatment

Trust in doctor	*n*	Mean (the Anderson Trust in Physician Scale scores) ± SD	*P* value
Adherence	166	43.97 ± 6.05	**0.0001**
Non-adherence	218	38.12 ± 5.41	
Total patients	384	-	

The mean given is the mean of the Anderson Trust in Physician Scale (TPS) with 11 questions, each rated on a 5-point Likert scale

## Discussion

Systemic treatment is increasingly used for patients with liver cancer. Numerous studies have demonstrated the significance and effectiveness of targeted therapies in patients with liver cancer, including those with advanced-stage disease [18–21]. Patients prescribed TKI drugs were typically in the high burden of tumors or advanced stages of the disease, with an ECOG score of 0 or 1, and good liver function (Child-Pugh A). Therefore, patients using Sorafenib and Lenvatinib as the first line or Regorafenib as the second line might have a similar status of liver cancer, including liver function and cancer stages. However, practical effectiveness largely depends on the patient’s adherence to treatment [22]. Previous studies have identified factors associated with nonadherence to medicine [23]. Our data contribute to the rate of nonadherence to standard oral cancer chemotherapy (OCT), including Sorafenib, Lenvatinib, and Regorafenib, and these factors significantly induced nonadherence in Vietnam. In Vietnam, these targeted therapies receive partial support from insurance or pharmaceutical companies, yet they still pose a significant economic burden on patients. Moreover, these medications have numerous reported side effects from various clinical trials, and whether these honestly act as barriers to treatment adherence is a question our research seeks to answer. Additionally, the patient’s awareness and attitude towards continued treatment in the advanced stage and the influencing factors in their surroundings may also contribute to the difficulty in proper medication use. Finally, the low level of trust in doctors or the connection with healthcare providers in Vietnam is a genuine concern for disease management, not only for cancer but also for other health conditions. Our study aims to explore these aspects concerning the non-adherence of HCC patients to oral cancer medications.

We surveyed 384 patients with HCC who received oral targeted therapy. Our results showed that 54.8% of all patients were nonadherent, with mean Morisky scores of 5,19 ± 0,52, even though the adherers achieved low mean Morisky scores (6.82 ± 0,45) (Table 2). The rate of nonadherence to medication in our study was higher than that in other populations, such as Japan (43.6%) [7], Spain (48%) [8] and Greece (20–30%) [24]. Therefore, the prevalence of nonadherence to medication was not consistent among the populations. The different study subjects with individual expectations, psychosocial characteristics, various cancer types and treatments, and healthcare insurance systems might induce this difference. On the other hand, patients included in the study were either new to taking oral TKIs or had been using TKIs previously. This could potentially introduce heterogeneity into the study population. However, there is currently no evidence indicating differences in treatment adherence between these two groups. When stratified into subgroups (new TKI users and ongoing TKI users), our analysis did not reveal significant differences in treatment adherence to TKIs. This suggests that the factors analyzed in the study are independent of the patient’s TKI usage duration and influence treatment non-adherence regardless of the time spent on TKI therapy.

Earlier studies have shown that the proportion of nonadherence was dependent on side effects but not on the regimens or doses of drugs [22, 23, 25, 26]. Our study yielded corresponding results. Our HCC patients experienced many side effects of drugs, such as fatigue (72.4%), followed by hand-foot syndrome, diarrhea, nausea and vomiting, insomnia, and abdominal pain (42.7%, 38.3%, 25%, 24.7%, and 12%, respectively (Fig. 2). All these side effects are common in patients treated with TKIs and have been reported at variable rates [27, 28]. However, we found that the proportion of side effects was noticeably higher among nonadherers than adherers (Table 4), indicating that side effects might induce nonadherent treatment. Discontinuous treatment might be due to patients voluntarily stopping to cope with side effects. Moreover, we found that nonadherers complained more about their concerns about the side effects of medicine than adherers (OR = 2.01, 95% CI = 1.36–3.1, *P* = 0.001, Table 6). Previous studies have shown that treatment-emergent adverse events prompt humans to interrupt medication intake [7, 25, 26]. In addition, our data were concordant with those of previous studies in that we did not find a relationship between the regimens of oral drugs or the dose of drugs for cancer and the rate of nonadherence. In Vietnam, we initiate the maximum dosage from the beginning, following recommendations from pharmaceutical companies and studies on the effectiveness of TKIs. The medication dosage used to analyze treatment non-adherence was the amount of TKI medication the patient took, recorded at the baseline. This can be the initial dose for newly prescribed TKIs or the adjusted dose for patients already undergoing treatment. Patients are instructed on lifestyle methods such as avoiding heavy labor, wearing comfortable shoes, daily blood pressure monitoring, and using supportive skin creams. When patients experience medication side effects, they are guided on monitoring and alleviating discomfort symptoms. Additionally, strategies for dose reduction or temporary discontinuation of TKIs are provided if patients communicate with their treating physicians. However, many patients choose only to follow these instructions, self-discontinuing or reducing medication doses after seeking medical advice. They may only reconnect with their doctors once side effects have subsided to seek guidance on continuing or potentially discontinuing treatment. Therefore, patients with HCC must receive helpful advice for all side effects of drugs from doctors, which can help reduce the rate of nonadherence.

Many psychosocial characteristics were strongly linked to nonadherence. Although a patient’s occupation and education were not correlated with a lack of adherence in our study, financial status directly affected the continuous treatment of patients. We found that the nonadherers had significantly higher out-of-pocket costs than the adherers (OR = 2.29, 95% CI = 1.32–3.98, *P* = 0.003). At the same time, they received meaningfully less copayment from their relatives or friends/neighbors than the discordant persons (*P* < 0.05). Our data are compatible with previous data [29–31], confirming that this underscores the persistent concern about financial matters that patients with cancer often face. Although insurance or pharmaceutical companies provide some financial assistance for these targeted therapies in Vietnam, they still represent a substantial economic burden for patients.

Other factors, such as insufficient awareness, knowledge, and understanding of patients, may be barriers to adherence to treatment. Patients with inadequate information about their clinical course, treatment, and drugs were more frequently classified into the nonadherent group than into the adherent group (Table 7).

Following those trends, our results indicated that nonadherent individuals had a significantly lower level of trust in doctors, with a mean Anderson Trust in Physician Scale score of 38.12 ± 5.41, compared to adherent individuals, with a mean Anderson Trust in Physician Scale score of 43.97 ± 6.05 (*P* = 0.0001). As a result, the poorer the connection between patients and doctors, the more insufficient the information that patients update. This might promote an increased rate of nonadherence to treatment.

Therefore, healthcare staff need to interact closely with patients to understand their concerns, the side effects of medications, or the symptoms of their condition. Through this understanding, they can provide detailed counseling and instructions to each patient, helping them feel at ease and remember to take medication according to their prescription. Moreover, to improve medication nonadherence and the level of trust in treating physicians, doctors need to inquire and connect with patients more regularly. Through this, they can provide meticulous advice regarding the patient’s condition, prescribe medications that fit their lifestyle and daily routines, and provide instructions for easier medication use. The advice may include suggestions such as placing medications in a visible location or requesting family members to remind the patient to take medication fully and on time.

This study has several limitations. First, we did not evaluate the specific timing and schedule of the administration of each medication. The nonadherence rates may be related to the timing of medication intake. If the timing of drugs does not align well with the patient’s daily routine, they may forget to take the medication. Second, this study relied on self-reporting, which is convenient and minimizes the burden on the patients. However, it introduces the risk of recall bias and social desirability bias, which can lead to inaccurate predictions of medication nonadherence. Third,

The severity of adverse events significantly impacts the patient’s quality of life and is a significant reason for nonadherence, but my study lacked this data. Last, the study did not assess medication nonadherence according to the stage. Patients who take medication for a longer duration may have a higher risk of nonadherence than those who have a shorter duration of medication use. This can lead to biased estimations of medication nonadherence.

In the future, adherence patterns should be considered to investigate the long-term adherence patterns of liver cancer patients to oral targeted therapies, analyze how adherence evolves over an extended treatment duration, and identify factors influencing sustained adherence. Moreover, intervention strategies might be investigated to explore and evaluate interventions to improve adherence to oral cancer medications. This might include educational programs, counseling, or technology to support and monitor patient adherence.

In conclusion, the rate of nonadherence to oral antineoplastic therapy in Vietnamese outpatients with HCC was high (54.8%). Adverse drug effects, patient anxiety regarding these side effects, financial constraints, limited knowledge about the medical condition and treatment regimen, and low trust in doctors are significant factors driving noncompliance with treatment. Enhancing oversight, providing clear medication guidance, implementing appropriate dosage adjustments, and offering comprehensive patient education are potential approaches to address nonadherence.

## Data Availability

The datasets generated and/or analyzed during the current study are not publicly available due to the privacy policy of the Vietnam military hospital but are available from the corresponding author upon reasonable request.
